# Predictors of radiation-induced hypothyroidism in nasopharyngeal carcinoma survivors after intensity-modulated radiotherapy

**DOI:** 10.1186/s13014-022-02028-z

**Published:** 2022-03-21

**Authors:** Ruiping Zhai, Yingchen Lyu, Mengshan Ni, Fangfang Kong, Chengrun Du, Chaosu Hu, Hongmei Ying

**Affiliations:** 1 Department of Radiation Oncology, Fudan University Shanghai Cancer Center, Room 703, Building 1, Dong’an Road 270, Shanghai, 200032 China; 2 Department of Oncology, Shanghai Medical College, Fudan University, Shanghai, 200032 China

**Keywords:** Hypothyroidism, Intensity-modulated radiotherapy, Nasopharyngeal carcinoma, Pituitary, Thyroid

## Abstract

**Background:**

The aim of the study is to identify clinical and dosimetric factors that could predict the risk of hypothyroidism in nasopharyngeal carcinoma (NPC) patients following intensity-modulated radiotherapy (IMRT).

**Methods:**

A total of 404 non-metastatic NPC patients were included in our study. All patients were treated with IMRT. The thyroid function were performed for all patients before and after radiation at regular intervals. The time onset for developing hypothyroidism was defined as the time interval between the completion of RT and the first recorded abnormal thyroid hormone test. The cumulative incidence rates of hypothyroidism were estimated using Kaplan–Meier method. Univariate and multivariate Cox regression analyses were performed to detect the most promising factors that were associated with hypothyroidism.

**Results:**

Median follow up was 60.6 months. The 3-, 5- and 7- year cumulative incidence rate of hypothyroidism was 39.4%, 49.1% and 54.7%, respectively. The median time to primary hypothyroidism and central hypothyroidism were 15.4 months (range 2.9–83.8 months) and 29.9 months (range 19.8–93.6 months), respectively. Univariate and multivariate analyses revealed that younger age, female gender and small thyroid volume were the most important factors in predicting the risk of hypothyroidism. Dtmean (mean dose of thyroid), V30-V50 (percentage of thyroid volume receiving a certain dose level) and VS45-VS60 (the absolute volumes of thyroid spared from various dose levels) remained statistically significant in multivariate analyses. Cutoff points of 45 Gy (Dtmean), 80% (Vt40) and 5 cm^3^ (VS45Gy) were identified to classify patients as high-risk or low-risk group.

**Conclusion:**

Thyroid Vt40 highly predicted the risk of hypothyroidism after IMRT for NPC patients. We recommended plan optimization objectives to reduce thyroid Vt40 to 80%.

*Trial registration*: Retrospectively registered.

## Background

The outcome of nasopharyngeal carcinoma (NPC) patients has improved dramatically over the decades by extensive application of intensity modulated radiotherapy (IMRT), with long-term survival now expected for most patients. Yet, even in the IMRT era, NPC survivors still face long-term sequelae which negatively affect their quality of life [1]. Hypothyroidism (HT), as one of the most common late toxicities, has been reported to occur in 40%-50% of patients who were treated with neck irradiation [2–4]. The most common symptoms of hypothyroidism are generally mild and tolerable, so they are often overlooked. However, if left untreated, hypothyroidism can induce cardiac and cognitive dysfunction and depression in the long run [5].

Primary hypothyroidism may develop as a result of radiation damage to the thyroid gland. Although the sequelae of primary hypothyroidism following radiation has been well documented, the published literature mainly focus on Hodgkin lymphoma (HL) and head and neck cancers (HNSCC) [4, 6–8]. However, NPC is distinct from HL and HNSCC in terms of radiation dose and treatment strategies. Generally, patients with HL are often prescribed with lower RT doses while patients with HNSCC are treated with surgical intervention and intensive radiotherapy. Thus, the heterogeneity in patients selection limits the generalizability of these studies to NPC patients. There is a need to scrutinize the rate and risk factors of radiation-induced hypothyroidism among NPC survivors.

Many related studies have suggested a dose-dependent risk of radiation-induced hypothyroidism, but the thresholds of dose-volume constraints to the thyroid gland varied. According to the published data, 30 Gy was identified to be predictive of hypothyroidism risk in HL patients, while for patients with HNSCC, the thresholds were set between 40 and 50 Gy [3, 6, 9]. Recently, several normal tissue complication probability (NTCP) models of thyroid based on non-NPC patients were developed which might offer us some dose-constraints for a clinically acceptable risk [10–12]. However, these models seems not applicable for NPC patients due to consideration of the possibility of central hypothyroidism among NPC patients [13, 14]. Luo et al. found a decreasing performance in their study when NPC patients were included in the external cohort [13]. Fan et al. further confirmed that the risk of hypothyroidism in NPC patients is more than twice that of the HNC cohort (without thyroidectomy) [14].

Compared with primary hypothyroidism, central hypothyroidism is considered less common for patients with NPC [4, 8, 13, 15]. However, due to adjacent anatomical location of pituitary gland, which is situated superior to the nasopharynx, radiation-induced pituitary injury should not be neglected. Previous studies showed considerable variations in incidence rates of central hypothyroidism following cranial irradiation. The published incidence rates ranged from 3 to 30% among patients with NPC and nonpituitary tumors [8, 15, 16]. Evidence suggested that the incidence of central hypothyroidism is significantly related to the total radiation dose but the dose-volume effect was less clear [4]. A number of studies suggested a threshold dose of 50 Gy for pituitary-hypothalamus hypothyroidism [4]. It is reported that the incidence rates were 3–6% when the radiation doses were lower than 50 Gy, and they were up to 14.9%-30% when the doses were as high as 50–70 Gy [17]. Also, the risk of central hypothyroidism has been reported to be associated with duration of follow-up after radiotherapy and methodological differences of endocrine evaluation [17]. Regarding central hypothyroidism among NPC patients, existing researches are retrospective, cross-sectional and lack accurate dosimetric parameters. Thus, prospective studies with homogeneous population, larger sample sizes and longer follow-up are needed.

Therefore, the purpose of this study was to investigate the long-term rate and risk factors for developing primary or central hypothyroidism in NPC patients after IMRT. Furthermore, we attempted to determine some useful dose constraints for plan optimization.

## Methods

### Patient selection

The study was an update of our previous research [18]. Details of the interim analysis has been published. Briefly, the eligibility criteria were patients newly diagnosed with biopsy-confirmed World Health Organization type 2 or 3 NPC; with an Eastern Cooperative Oncology Group performance status of 0 to 1; with no distant metastasis and normal thyroid function. Patients who had previous pituitary/thyroid disorders or related treatment including thyroid surgery, irradiation to the head and neck/pituitary area were not included. The trial was approved by the institutional review board of our hospital and all patients provided written informed consent for inclusion in the study.

### Treatment planning

Patients with stage T1-T2 N0M0 disease were treated with the radical RT alone. Concurrent single agent chemotherapy in combination with radiotherapy was given to those with stage T1-T2 N1M0 disease. Patients with stage III-IVB disease were treated with comprehensive treatment plan, including radiation and cisplatin-based chemotherapy in a concurrent and/or sequential sequence.

The radiation treatment plan of the trial has been described in details previously [18]. Patients were immobilized in the supine treatment position with a thermoplastic device that covered head and neck area and the shoulders. Contrast-enhanced planning computed tomography (CT) scans with a 5-mm slide thickness were obtained for every patient. Image fusion using diagnostic MRI and RT planning CT images were performed for all patients to accurately delineate tumor and organs at risk (OAR).

The gross tumor volume (GTV) included the primary tumor of nasopharynx (GTV-P) and the pathologic lymph nodes (GTV-LN). The clinical target volume at high risk (CTV1) included GTV-P with a 5 mm margin, the whole nasopharynx, the parapharyngeal spaces, the base of skull, the pterygoid plates, the anterior half or two thirds of the clivus (the whole clivus was covered if the clivus was involved), lower half of sphenoid sinuses (the whole was involved for T3-T4 lesions), petrous tips, the posterior of the nasal cavities, the maxillary sinuses and lymphatic regions. The CTV1 at high risk for neck area included bilateral retropharyngeal nodes, level II, level III and VA lymph nodes area, and the station of the involved lymph nodes. The clinical target volume at low risk (CTV2) covered the level IV and level VB (in case that no positive lymph nodes were found in the lower neck). Selective sparing of level IV and level VB was performed in stage N0 patients. The planning tumor volume (PTV-G, PTV-N and PTV-C-1, PTV-C-2) was generated by expanding the corresponding volumes of GTV and CTV by 3–5 mm, respectively.

The dose prescription was relatively uniform throughout the PTV in our series. Simultaneous integrated boost (SIB) technique was adopted to deliver different dose levels to different target volume within a single treatment fraction. The doses prescribed for PTV-G were 66 Gy in 30 fractions for T1-T2 lesions and 70.4 Gy in 32 fractions for T3-T4 lesions. The corresponding dose prescribed for PTV-LN was 66 Gy in 30 or 32 fractions. PTV-CTV1 was prescribed 60 Gy and PTV-CTV2 was prescribed 54 Gy in 30 or 32 fractions, respectively.

For each PTV at each dose level, the plan generated aimed to cover at least 95% of the volume of PTV with the planned dose while keeping the maximum dose below 110% of the prescribed dose in or out of PTV. The plan optimization also included the dose limits for organs at risk. The maximum doses were 54 Gy for the brain stem, optic nerves and optic chiasm, 45 Gy for the spinal cord, 60 Gy for the temporal lobe, 8 Gy for lens, and 26 Gy for the parotid gland mean dose.

### Gland delineation and dosimetry parameters

The pituitary and the thyroid glands were contoured manually by an experienced radiation physician for all the patients on the CT simulation images. We did not give specific dose constraints to both glands due to the considerations to optimize coverage of the tumor. All IMRT plans were done using the Pinnacle TPS (version 9.0, Philips Radiation Oncology Systems, Fitchburg, WI). Cumulative dose-volume histograms (DVH) were calculated from the planning system. The following dose–volume statistics were obtained from the DVH: the doses to the pituitary gland (mean, Dpmean; minimum, Dpmin; maximum, Dpmax); the absolute pituitary and thyroid gland volume (Vp and Vt); the doses to the thyroid gland (mean, Dtmean; minimum, Dtmin; maximum, Dtmax); the percentage of thyroid volume receiving more than 30 Gy (Vt30), Vt40, Vt45, Vt50 and Vt60; the percentage of pituitary volume receiving more than 50 Gy (Vp50), Vp55, Vp60, Vp70; the absolute volume of the thyroid spared from various dose levels including 45 Gy (VS45), VS50, VS55 and VS 60.

### Thyroid function evaluation

The thyroid function including thyroid stimulating hormone (TSH), free thyroxine (FT4), free triiodothyronine (FT3), thyroid peroxidase antibody (TPO) and thyroglobulin antibody (TGAb) measurements were performed for all patients before radiation, at the end of radiotherapy and then in 3–6 months intervals for the first two years. Thereafter, follow-up visits were scheduled every 6 months from the third to the fifth year and then yearly afterwards. Clinical assessments were also scheduled at the same time on a regular basis or as needed clinically. The Assay Kit of TSH and FT4 has been described in detail previously [18]. Transient thyrotoxicosis was identified as a low serum TSH level with or without an elevated FT4 or FT3 level. We defined primary hypothyroidism as an elevated TSH serum level (> 4.94 mIU/L) in combination with a normal or low serum FT4 level, regardless of symptoms. Central hypothyroidism was identified as a low serum FT4 level (< 9.01 pmol/L) and non-elevated TSH level. The time onset for developing hypothyroidism was defined as the time interval between the completion of RT and the first abnormal thyroid hormone test indicating the presence of hypothyroidism.

### Statistical analysis

All the statistical analyses were carried out using SPSS version 26 (SPSS Inc., Chicago, IL). The cumulative incidence rates of hypothyroidism were estimated using Kaplan–Meier method, and log-rank test to evaluate the difference between groups. Univariate cox proportional hazard models were performed to detect which clinical and dosimetric parameters were associated with the development of hypothyroidism at 5 years post RT. The following variables were evaluated: age, sex, receipt of chemotherapy, neck surgery, thyrotoxicosis, T and N classifications, UICC 2010 stage, the volume of the thyroid and pituitary glands and multiple dose parameters of both pituitary and thyroid glands. Multivariate analyses were performed on variables with *p* < 0.05 from the univariate analyses. Pearson correlation analyses were used to examine the association between two variables to avoid the influence of collinearity. Dosimetric parameters were added to the multivariate model separately at a time in model fitting. Optimal threshold analyses of the dosimetric parameters in the multivariate models were conducted using Receiver Operating Characteristics (ROC) curve analyses. All statistical tests were two-sided and a *p*-value of < 0.05 was considered statistically significant.

## Results

Seven hundred and ten patients diagnosed with non-metastatic NPC between February 2012 to December 2016 in Fudan University Shanghai Cancer Center were eligible. We excluded 43 (6%) who did not receive radiotherapy in our institution, 33 (5%) who underwent thyroid surgery or had abnormal TSH levels, 29 (4%) who had metastatic diseases (stage IVC), 53 (7%) who experienced tumor progression within one year after radiotherapy, and 148 (20%) who had their thyroid hormones tested in local hospitals but no record could be obtained. The final study population therefore consisted of 404 patients with non-metastatic NPC. In this data set, the majority of patients had shown good compliance by the last follow up. A total of 341 patients (84.4%) were followed more than 3 years, 292 patients (72.2%) were followed more than 4 years, and 52.9% of the study population were followed more than 5 years. The median follow-up was 60.6 months (range 15.4–104.6 months). The median age of all patients was 48 years (range 18–70 years), with 301 (74.5%) males and 103 (25.5%) females. The baseline characteristics of patients are shown in Table 1.

**Table 1:** Baseline characteristics of the 404 NPC patients receiving IMRT

Variable	Number	%
Sex		
Male	301	74.5
Female	103	25.5
Age, years		
Median		
< 48 y	200	49.5
≥ 48 y	204	50.5
Thyroid volume, cm^3^		
Median		
< 16 cm^3^	200	49.5
≥ 16 cm^3^	204	50.5
T-category		
T1	35	8.7
T2	141	34.9
T3	139	34.4
T4	89	22.0
N-category		
N0	19	4.7
N1	110	27.2
N2	151	37.4
N3	124	30.7
UICC stage		
I	5	1.2
II	61	15.1
III	146	36.1
IV	192	47.5
Chemotherapy		
Induction	311	77.0
Concurrent	73	18.0
Adjuvant	146	36.1
Neck surgery		
Yes	23	5.7
No	381	94.3

UICC, Union for International Cancer Control, 2010 Edition

**Fig. 1:**
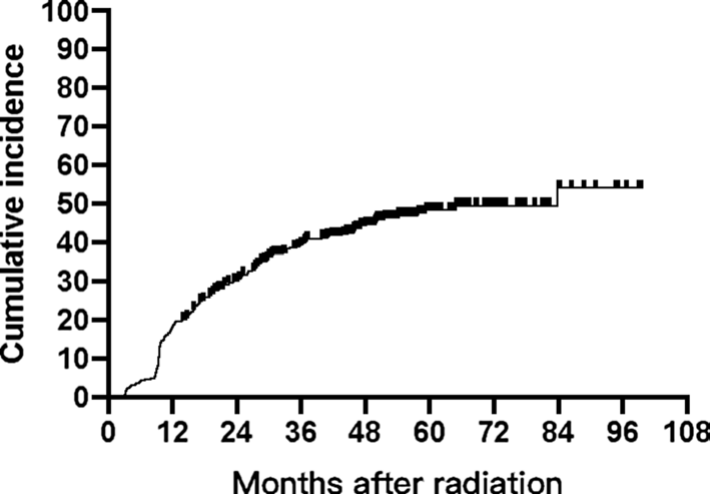
Cumulative incidence curve of radiation-induced hypothyroidism in 404 NPC patients

Transient thyrotoxicosis was observed in 36.9% (149/404) of the patients at the end of radiotherapy. However, nearly all of them returned to euthyroid state spontaneously 3–6 months later. During the follow up period, a total of 181 (44.8%) patients were diagnosed with hypothyroidism, in which173 had primary hypothyroidism, 6 had central hypothyroidism and 2 were with a mixture of both types. The 3-, 5- and 7- year cumulative incidence rates of hypothyroidism were 39.4%, 49.1% and 54.7%, respectively (Fig. 1). The median time to primary hypothyroidism and central hypothyroidism were 15.4 months (range 2.9–83.8 months) and 29.9 months (range 19.8–93.6 months), respectively.

Univariate analyses indicated that younger age, female gender, advanced N-stage, use of chemotherapy, the condition of thyrotoxicosis and smaller thyroid volume were associated with a higher risk of hypothyroidism (Table 2). Nearly all the dosimetric parameters of the thyroid gland (except Dmax and Vt60) were found to significantly affect the development of hypothyroidism (Table 3). However, none of the pituitary-related dose parameters reached statistical significance (Table 3).

**Table 2:** Univariate analyses with respect to clinical factors and the risk of HT

Variable	Univariate analysis	
	HR (95% CI)	*p* value
Sex		
Male		
Female	1.999 (1.468–2.722)	0.000*
Age		
Per year increase	0.976 (0.964–0.988)	0.000*
Chemotherapy		
Yes		
No	1.587 (1.024–2.459)	0.039*
Neck surgery		
Yes		
No	1.306 (0.727–2.347)	0.371
Thyrotoxicosis		
Yes		
No	0.609 (0.440–0.844)	0.003*
T classification		
T1–T2		
T3–T4	1.308 (0.968–1.768)	0.08
N classification		
N0–N1		
N2–N3	1.524 (1.086–2.137)	0.015*
UICC 2010 stage		
I–II		
III–IV	1.016 (0.679–1.522)	0.937
Thyroid volume, per cm^3^ increase	0.905 (0.877–0.934)	0.000*
Pituitary volume, per cm^3^ increase	0.870 (0.634–1.192)	0.386

*Means *p* < 0.05

**Table 3:** Univariate analyses with respect to dosimetric factors and the risk of HT

Variable	Univariate analyses	*p* value
	HR (95% CI)	
Dtmean, per Gy increase	1.000 (1.000–1.001)	0.000*
Dtmin, per Gy increase	1.000 (1.000–1.000)	0.000*
Dtmax, per Gy increase	1.000 (1.000–1.000)	0.551
Thyroid Vt30, per % increase	7.735 (2.648–22.594)	0.000*
Thyroid Vt40, per % increase	7.394 (2.979–18.353)	0.000*
Thyroid Vt45, per % increase	7.544 (3.054–18.639)	0.000*
Thyroid Vt50, per % increase	7.042 (2.781–17.832)	0.000*
Thyroid Vt60, per % increase	0.901 (0.225–3.601)	0.883
Dpmin, per Gy increase	1.000 (1.000–1.000)	0.360
Dpmean, per Gy increase	1.000 (1.000–1.000)	0.391
Dpmax, per Gy increase	1.000 (1.000–1.000)	0.979
Pituitary Vp50, per % increase	1.136 (0.708–1.822)	0.596
Thyroid VS45, per cm^3^ increase	0.855 (0.815–0.898)	0.000*
Dichotomous (< 5cm^3^ vs ≥ 5cm^3^)	0.474 (0.352–0.639)	0.000*
Thyroid VS50, per cm^3^ increase	0.863 (0.827–0.902)	0.000*
Dichotomous (< 8cm^3^ vs ≥ 8cm^3^)	0.445 (0.326–0.607)	0.000*
Thyroid VS55, per cm^3^ increase	0.886 (0.855–0.918)	0.000*
Dichotomous (< 12cm^3^ vs ≥ 12cm^3^)	0.437 (0.318–0.601)	0.000*
Thyroid VS60, per cm^3^ increase	0.904 (0.877–0.932)	0.000*
Dichotomous (< 15cm^3^ vs ≥ 15cm^3^)	0.482 (0.354–0.655)	0.000*

Thyroid Vt30, Vt40, Vt45, Vt50 and Vt60 = percentages of thyroid volume receiving 30 Gy, 40 Gy, 45 Gy, 50 Gy and 60 Gy; Vp50 = percentage of pituitary volume receiving 50 Gy; Thyroid VS 45, 50, 55 and 60 = spared volume of thyroid receiving 45 Gy, 50 Gy, 55 Gy and 60 Gy

*Means *p* < 0.05

**Fig. 2:**
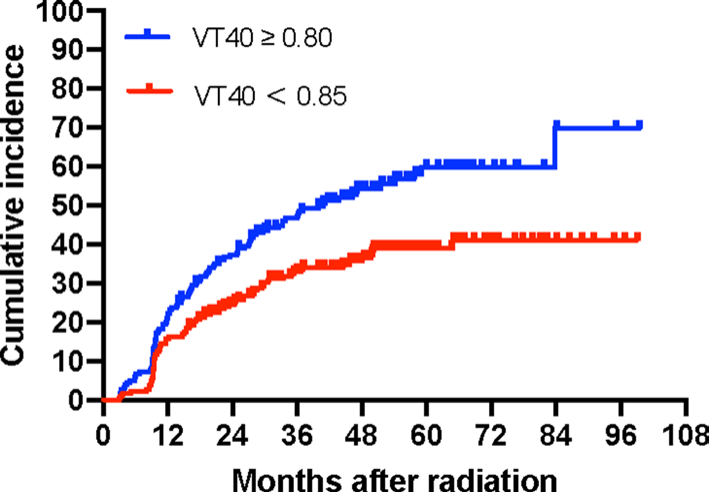
Cumulative incidence curve of hypothyroidism by V40 with a cut-off of 80%

In multivariate analyses, younger age, female gender and small thyroid volume remained the most important factors in predicting the risk of hypothyroidism. Furthermore, thyrotoxicosis was identified to be a protective factor but with limited evidence. Chemotherapy failed to show an association with the risk of hypothyroidism after adjustment for other variables. The updated results of our study further confirmed a clear dose-dependent relationship for hypothyroidism development: Dtmean (*p* = 0.001), Vt30 (*p* = 0.002), Vt40 (*p* = 0.002), Vt45 (*p* = 0.003) and Vt50 (*p* = 0.002) of the thyroid were all identified to be promising variables (Table 4). With regard to ROC analyses of the dosimetric parameters, the area under the curve (AUC) was significantly different from 0.05 for all the above parameters, and the difference was greatest for V40 (AUC = 0.631, *p* = 0.000). A threshold of 80% for V40 was identified to classify patients into high-risk and low-risk groups for the development of hypothyroidism. The incidence rates of hypothyroidism in the group with V40 < 80% and V40 ≥ 80% were 36.3% and 54.7%, respectively (Fig. 2).

**Table 4:** Multivariate analyses with respect to factors and the risk of HT

Variable	Multivariate analyses	
	HR (95% CI)	*p* value
The Dtmean model		
Age (per year increase)	0.981 (0.968–0.993)	0.002*
Female (vs male)	1.551 (1.121–2.146)	0.008*
Chemotherapy (vs no)	1.192 (0.747–1.902)	0.461
Thyrotoxicosis (vs no)	0.673 (0.481–0.943)	0.021*
Thyroid volume (per cm^3^ increase)	0.929 (0.899–0.960)	0.000*
Dtmean (per Gy increase)	1.000 (1.000–1.001)	0.001*
The Vt 30 model		
Age (per year increase)	0.981 (0.969–0.993)	0.002*
Female (vs male)	1.562 (1.130–2.519)	0.007*
Chemotherapy (vs no)	1.196 (0.746–1.917)	0.457
Thyrotoxicosis (vs no)	0.672 (0.480–0.941)	0.021*
Thyroid volume (per cm^3^ increase)	0.929 (0.899–0.960)	0.000*
Thyroid Vt30 (per % increase)	6.613 (1.968–19.304)	0.002*
The Vt 40 model		
Age (per year increase)	0.981 (0.969–0.994)	0.003*
Female (vs male)	1.550 (1.121–2.143)	0.008*
Chemotherapy (vs no)	1.246 (0.780–1.992)	0.357
Thyrotoxicosis (vs no)	0.684 (0.424–1.092)	0.027*
Thyroid volume (per cm^3^ increase)	0.931 (0.901–1.963)	0.000*
Thyroid Vt40 (per % increase)	4.411 (1.732–11.233)	0.002*
The Vt 50 Model		
Age (per year increase)	0.981 (0.968–0.993)	0.002*
Female (vs male)	1.568 (1.134–2.169)	0.006*
Chemotherapy (vs no)	1.332 (0.841–2.112)	0.222
Thyrotoxicosis (vs no)	0.686 (0.490–0.960)	0.028*
Thyroid volume (per cm^3^ increase)	0.930 (0.900–0.961)	0.000*
Thyroid Vt50 (per % increase)	4.689 (1.747–12.591)	0.002*
The Vt 45 Model		
Age (per year increase)	0.981 (0.969–0.993)	0.003*
Female (vs male)	1.559 (1.127–2.155)	0.007*
Chemotherapy (vs no)	1.294 (0.813–2.060)	0.277
Thyrotoxicosis (vs no)	0.688 (0.492–0.964)	0.030*
Thyroid volume (per cm^3^ increase)	0.932 (0.902–0.964)	0.000*
Thyroid Vt45 (per % increase)	4.158 (1.639–10.547)	0.003*

^*^Means *p* < 0.05

**Fig. 3:**
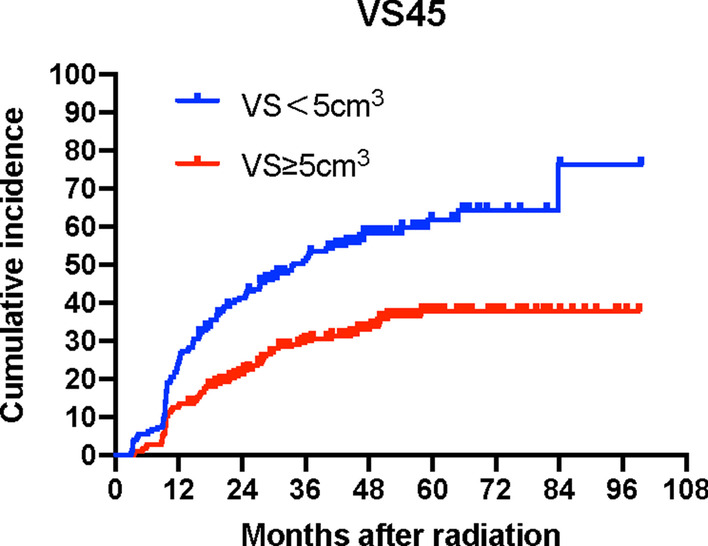
Cumulative incidence curve of hypothyroidism by VS45 with a cut-off of 5 cm^3^

Likewise, the absolute volumes of thyroid spared from various dose levels were also significant factors in multivariate analyses: VS45 Gy (*p* = 0.000), VS50 Gy (*p* = 0.000), VS55 Gy (*p* = 0.000) and VS60 Gy (*p* = 0.000) (Table 5). The Kaplan–Meier curve for VS45 Gy with a threshold value of 5 cm^3^ is shown in Fig. 3. Log-rank tests revealed that patients whose VS45 Gy ≥ 5 cm^3^ had a five-year hypothyroidism rate of 37.7% as opposed to 68.6% for those whose VS45 Gy < 5 cm^3^ (*p* = 0.000).

**Table 5:** Multivariate analyses with respect to factors and the risk of HT

Variable	Multivariate analyses	
	HR (95% CI)	*p* value
The VS45 Model		
Age (per year increase)	0.979 (0.967–0.991)	0.001*
Female (vs male)	1.690 (1.231–2.319)	0.001*
Chemotherapy (vs no)	1.194 (0.758–1.881)	0.444
Thyrotoxicosis (vs no)	0.638 (0.459–0.887)	0.008*
Thyroid VS45 (per cm^3^ increase)	0.876 (0.833–0.920)	0.000*
The VS50 Model		
Age (per year increase)	0.979 (0.967–0.992)	0.001*
Female (vs male)	1.658 (1.207–2.278)	0.002*
Chemotherapy (vs no)	1.284 (0.818–2.015)	0.277
Thyrotoxicosis (vs no)	0.656 (0.472–0.913)	0.012*
Thyroid VS50 (per cm^3^ increase)	0.883 (0.844–0.924)	0.000*
The VS55 Model		
Age (per year increase)	0.981 (0.968–0.993)	0.002*
Female (vs male)	1.637 (1.190–2.250)	0.002*
Chemotherapy (vs no)	1.435 (0.918–2.242)	0.113
Thyrotoxicosis (vs no)	0.665 (0.478–0.926)	0.016*
Thyroid VS55 (per cm^3^ increase)	0.905 (0.871–0.940)	0.000*
The VS60 Model		
Age (per year increase)	0.982 (0.970–0.994)	0.004*
Female (vs male)	1.652 (1.203–2.268)	0.002*
Chemotherapy (vs no)	1.562 (1.002–2.435)	0.049
Thyrotoxicosis (vs no)	0.677 (0.485–0.944)	0.021*
Thyroid VS60 (per cm^3^ increase)	0.921 (0.891–0.951)	0.000*

*Means *p* < 0.05

## Discussion

Our findings suggest that hypothyroidism is a frequent complication following direct irradiation to the thyroid gland (more common) or the pituitary gland among NPC patients. The risk of hypothyroidism largely depends on the radiation dose delivered to each gland and the time relapsed after irradiation. The data presented here is a large data set that examined the relationship between various clinical and dosimetric parameters and the development of hypothyroidism among NPC patients after IMRT.

### The plateau for the development of primary hypothyroidism

The cumulative incidence rates were generally in accordance with previously published results, and showed significant time-effect dependence (with 39.4%, 48.4% and 54.1% at 3-, 5- and 7- years of follow-up time). We noticed that the incidence of hypothyroidism increased to a maximum within 2 to 3 years after treatment, then became steady afterwards and reached a plateau at about 5 years. Similar phenomenon was found in Bhandare et al.’s study, in which the incidence for subclinical primary hypothyroidism was 29% at 5 years and it did not increase by 10 years after treatment [8]. A 10-year latency for the plateau period was also reported by Wu et al. and her colleagues [16]. Studies conducted on HL patients also found clear support for the trend of hypothyroidism risk [7]. However, the latency period for HL patients was completely different. The largest study to date from Stanford by Hancock et al. indicated that the risk of hypothyroidism after irradiation increased only slightly from 43% at 20 years to 47% at 26 years [7]. Time effect is becoming less important with extended follow-up, especially after the “plateau period”. It is difficult to explain the great variations in the frequency and the length of latency time interval for the development of hypothyroidism. Lin et al.’s study showed us the pattern of radiation-induced thyroid gland changes in NPC patients: the mean thyroid volume followed a decreasing trend after RT, reaching a minimum at 30 months and slightly increased afterwards [19]. It is of interest to note that partial recovery in terms of thyroid volume along with thyroid function occurred after 30 months interval. However, it is still not clear whether the “plateau period” is caused by spontaneous recovery of thyroid function or sufficient active thyroid reserve. As the mechanism of radiation damage to the thyroid gland has not been well understood, longer monitoring and more researches are still needed to provide more information about this late sequelae.

### Reasons why central hypothyroidism was less common

Compared with primary hypothyroidism, central hypothyroidism due to intensive cranial irradiation is thought to be less common and occurs after a relatively longer latency period [8, 20, 21]. According to several recent reports, the overall incidence of TSH deficiency was about 5.4–14% with a median follow-up of 3.6–8 years in survivors with head and nasopharyngeal tumors [8, 22, 23]. Ratnasingam and colleagues reported the median time elapsed from treatment for patients who had hypothalamic-pituitary dysfunction was 9 years (range 7–21) [24]. In their data, the minimum duration from therapy was 7 years, and the latency period prolonged to almost 21 years. A recent meta-analysis of 18 studies with a total of 813 patients with NPC and non-pituitary brain tumor showed a prevalence of 0.25 (95% CI: 0.16–0.37) [25]. In the present study, only 2% of the patients (n = 7) were identified with central hypothyroidism, which is much lower than previous reports. There are several possible reasons. Firstly, the radioresistant characteristic of the TSH axis is the most important reason. It has been demonstrated that GH axis is the most sensitive followed by the LH/FSH, adrenocorticotropic-hormone (ACTH) and TSH axes [17, 22]. Based on temporal presentation and frequency of occurrence, it is not hard to understand the less frequency and longer latency of central hypothyroidism. Secondly, the risk of TSH deficiency is determined by the radiation dose delivered to the hypothalamic-pituitary area. Littley et al. concluded that the total dose to the H-P axis is a major determinant of the speed of onset of central hypothyroidism [26]. Darzy et al. found the incidence is distinctly high in patients with radiation doses in excess of 60 Gy. In their study, the frequency of central hypothyroidism was as low as 3%-9% with doses of 30–50 Gy and reached up to 30%-60% with doses greater than 60 Gy after 10 years [17]. In the present study, the median value of Dpmean was 50.4 Gy, which was far below the recommendation of D_0.03 cc_ dose ≤ 60 Gy in the updated guideline of QUANTEC (Quantitative analysis of normal tissue effects in the clinic, QUANTEC) [27]. The possible reason for the relatively lower dose is that only 20.2% of the patients were diagnosed with T4 disease. Thirdly, the low incidence could be attributed in part to decreased sensitivity of testing methodologies. The diagnosis of central hypothyroidism is based on low circulating levels of FT4 in the presence of low to normal TSH concentrations in our study. However, what should be noted is TSH level sometimes elevates in the state of mixed hypothyroidism or latent central hypothyroidism due to the secretion of biologically inactive TSH [28]. This situation in fact mirrors that observed in primary hypothyroidism and might lead to an underestimate of central hypothyroidism risk. Also, the wide reference range for FT4 often makes diagnosis of central hypothyroidism challenging. Alexopoulou et al. suggested a greater than 20% decrease in circulating levels of FT4 was indicated of central hypothyroidism, even if serum concentrations of FT4 are still in the normal range [17]. Rose et al. and their colleagues outlined similar observation [29]. Some authors suggested a progressive decline in FT4 serum level albeit still within the normal range was indicative of central hypothyroidism [17]. Other investigators have recommended the TSH surge test and the TRH test to be required for the diagnosis of central hypothyroidism [17]. Although debatable, these testing standards made a positive diagnosis for central hypothyroidism difficult.

### Clinical factors associated with hypothyroidism occurrence

The major clinical risk factors for hypothyroidism identified in this study were younger age, female sex, thyrotoxicosis and smaller thyroid volume. Multivariate analyses indicated that the relative risk of hypothyroidism decreased by 0.98 with every additional year of age, and decreased by 0.93 with per unit increase in thyroid volume. Our study also demonstrated that women had a higher risk of developing hypothyroidism, with a 1.6:1 female/male ratio. The effects of age and female gender were in good agreement with the findings reported by Hancock et al. [7]. In the well-documented largest study conducted in HL patients, Hancock et al. and their colleagues found that younger age (RR: 1:0.99) and female sex (RR: 1:1.60) were both correlated with an increased risk for the development of hypothyroidism [30]. In the study by Diaz et al., the relative risk for increasing age and increasing thyroid volume were 0.93 and 0.96, respectively [31]. Fan et al. examined the risk factors among 14,893 NPC patients and 16,105 HNC patients, and their data further demonstrated the significant influence of younger age and female sex [14]. However, inconsistent results regarding the effect of age and sex were also reported. Diaz et al. and Wu et al. found no effect of female gender on the development of hypothyroidism [16, 31]. They argued that women had smaller thyroid volume than men, and the sex effect may partly be confounded with the differences in thyroid volumes. In the literature-based meta-analysis by Vogelius et al., the risk in women was 1.6 times than that in men while age didn’t appear to influence the risk of hypothyroidism [3]. Colevas et al. showed contrary results that increasing age was associated with a higher risk of hypothyroidism especially for those who were over 60 years old [32]. It should be noted that the elderly do have an increased prevalence of hypothyroidism among general population, although this trend has not been described in other studies. Also complex physiological alterations occur within the hypothalamo-pituitary-thyroid axis of aging population. It is difficult to distinguish these age-related changes from the pathophysiological alterations caused by radiation in the elderly.

Interestingly, our data indicated the relative risk of hypothyroidism decreased by 0.68 with the occurrence of transient thyrotoxicosis in multivariate analyses. Similar findings were not observed in other studies, despite the high frequency of thyrotoxicosis as an early response to irradiation [19]. The thyrotoxic phase was thought to be attributed to increased permeability of cellular membrane without destructive changes in the thyroid gland [33]. This is a good explanation for the spontaneous recovery from transient thyrotoxicosis observed in our series patients. However, we are not able to explain the protective effect of thyrotoxicosis in hypothyroidism occurrence, and further explorations with longer follow-up and larger sample size are still needed.

### Dosimetric factors associated with hypothyroidism occurrence

Our study demonstrated a dose-dependent risk of radiation-induced hypothyroidism. The thyroid volume and a series of dosimetric variables were identified to be significant factors in multivariate analyses. This is consistent with the results of several published multivariate normal tissue complication probability models (NTCP) for radiation-induced hypothyroidism [10–12]. Smaller thyroid volume was associated with higher risk of hypothyroidism in nearly all of the NTCP models. But the dosimetric variables were significantly different among different studies.

**Fig. 4:**
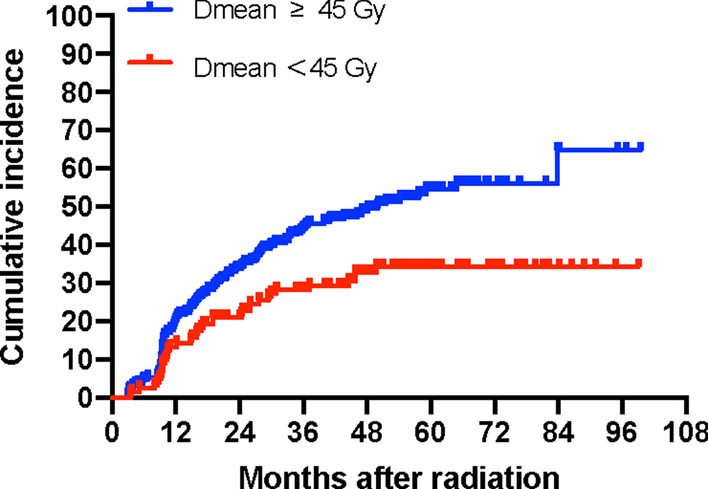
Cumulative incidence curve of hypothyroidism by Demean with a cut-off of 45 Gy

Many investigators suggested the mean dose of the thyroid as the most promising dosimetric factor. This is probably due to the parallel structure of the thyroid gland. Therefore, to keep the mean thyroid dose as low as possible could reasonably reduce the risk of hypothyroidism. Vogelius estimated a 50% risk of hypothyroidism at thyroid mean doses of 45 Gy [3]. Bhandare et al. showed the incidence could increase by 16% when the thyroid dose was above 45 Gy [8]. Bakhshandeh et al.’s mean dose model further confirmed the parallel architecture for the thyroid gland with a strong volume effect: D50 was suggested approximately 44 Gy [34]. Our conclusion was in good agreement with the above recommendation of 45 Gy. As is shown in Fig. 4, those with thyroid mean dose < 45 Gy had significantly lower rates of hypothyroidism than those with mean dose ≥ 45 Gy (31.9% vs 49.8%, *p* < 0.05). There are also other practical references for dose constraints of the thyroid. Fujiwara et al. suggested a more stringent mean dose of 30 Gy as the threshold [35]. And the recommended dose in the recently published international guideline was to aim for a mean dose of 50 Gy [27]. In the NTCP models by Rønjom et al., individual dose constraints were proposed: 26 Gy, 38 Gy, 48 Gy and 61 Gy for thyroid volumes of 10, 15, 20 and 25 cm^3^, respectively [12]. However, Rønjom et al. estimated a five-year risk of developing hypothyroidism of 26%, which was much lower than other reports. Due to the relatively lower incidence rate, the performance of the NTCP model needs more external validation before eventual implementation in clinical practice.

Irradiated volume of thyroid gland has been demonstrated as significant variables in a series of studies. But the clear dose-volume relationship has not been definitely determined. A variety of dose-volume constraints ranging from V25 to V50 were found to be significant factors for post-radiation hypothyroidism. Generally speaking, lower doses as V25-V30 (the percentage of the thyroid receiving 20 to 30 Gy) were significant risk factors for HL patients while V40-V45 were promising predictors for HNSCC patients [6, 9, 36–39]. It seems that HL patients are more susceptible to radiation-induced hypothyroidism than head and neck cancer patients. In the meta-analysis, Vogelius et al. noted the same tendency toward higher radiation-induced hypothyroidism risk in HL patients than in HNSCC patients receiving the same dose [3]. The strongest evidence was that a dose as low as 10 Gy could produce a risk of hypothyroidism in HL patients from the plot. These findings suggest the necessity to reduce the volume of thyroid receiving 30 Gy or less for NPC patients. Our conclusions were in consistent with previous constraints, V30-V50 (except V60) maintained the statistically significant association with the risk of hypothyroidism in multivariate analyses. This would imply that NPC patients may have the same dose thresholds as HL patients. However, it is difficult to define a threshold of V30 in our study as the prescribed dose to neck was 60 Gy for the majority of patients. The dose volumetric thresholds obtained from lymphoma studies as V25 < 63.5% or V30 < 62.5% may be difficult to apply to patients with NPC [6, 9]. We also identify an optimal cutoff of V40 < 80% from the ROC analysis, which was similar with the reports by Sommat et al. They identified V40 to be most predictive of hypothyroidism and suggested a threshold of V40 of 85% in their study of 102 patients with nasopharyngeal cancer [36]. Kim et al. suggested a more stringent threshold, where V45 lower than 50% was recommended in their study of 114 patients with head and neck cancer [39]. Yet, the results should be interpreted cautiously due to relatively shorter follow-up duration, high proportion of surgical intervention and lower AUC value. In conclusion, to minimize the risk of hypothyroidism after RT, it is of great importance to reduce the percentage of volume receiving a certain dose especially the dose of 40 Gy.

Other investigators have proposed the undisturbed thyroid volume spared from specified dose levels (VSxxGy) as significant factors. Pinnix et al. suggested a threshold thyroid volume of 2.2 mL should be spared from ≥ 25 Gy [6]. Chyan et al. revealed that patients with VS45 Gy of at least 3 cc, VS50 Gy of at least 5 cc could significantly lower the risk of hypothyroidism development [38]. Lee et al. recently suggested at least 10 cm^3^ of thyroid should be spared from doses exceeding 60 Gy and at least 5 cm^3^ be spared from doses exceeding 45 Gy to reduce the risk of hypothyroidism among NPC patients [40]. This principle of sparing an absolute volume of thyroid from injury to limit risk of hypothyroidism was also demonstrated in surgical series [6]. Our study suggested the same dose constraints that at least 5 cm^3^ should be spared from doses exceeding 45 Gy. All these findings provided us different dose limit targets. Some authors insisted the amount of thyroid follicular cells that are spared from radiation might be the most important determining factor [40]. This is probably because undisturbed thyroid volume is responsible for the production of thyroid hormones and represents thyroid hormone reserve. However, it must be cautioned that these dose limitation might not be applicable for all the situations especially when the lymph nodes were large-sized and the target volume must be expanded to achieve better target coverage.

## Conclusion

In summary, our data confirm a time-dependent and dose-dependent risk of radiation-induced hypothyroidism. Compared with central hypothyroidism, primary hypothyroidism occurs fairly common and has a considerably shorter latency period. Younger age, female sex and smaller thyroid volume are associated with increased risk of hypothyroidism after IMRT in NPC patients. Given the prevalence of hypothyroidism in our cohort, we recommend the use of IMRT treatment optimization objectives to restrict Dmean < 45 Gy, V40 < 80% or VS45 ≥ 5 cm^3^.

## Data Availability

Not applicable.

## Abbreviations

NPC: Nasopharyngeal carcinoma
IMRT: Intensity-modulated radiotherapy
TSH: Thyroid stimulating hormone
FT4: Free thyroxine
FT3: Free triiodothyronine
NTCP: Normal tissue complication probability models
Dtmean: Mean dose of thyroid
Vt30-Vt50: Percentage of thyroid volume receiving a certain dose level
VS45-VS60: The absolute volumes of thyroid spared from various dose levels
